# Technique for Robotic Stereotactic Irradiation of Choroidal Melanoma

**DOI:** 10.7759/cureus.582

**Published:** 2016-04-21

**Authors:** Dominic Béliveau-Nadeau, Sonia Callejo, David Roberge

**Affiliations:** 1 Department of Radiation Oncology, Centre hospitalier de l'université de Montréal (CHUM); 2 Department of Ophthalmology, Centre hospitalier de l'université de Montréal (CHUM); 3 Department of Oncology, Division of Radiation Oncology, McGill University Health Center; 4 Department of Radiology, Radiation Oncology and Nuclear Medicine, University of Montreal

**Keywords:** choroidal melanoma, cyberknife

## Abstract

Radiotherapy has a long history in the organ-sparing management of choroidal melanoma. Joining plaque radiotherapy and proton irradiation, stereotactic robotic photon irradiation is a new tool in the radiation oncologist’s armamentarium for ocular tumors. The non-coplanar fields with steep dose gradients are well suited to spare uninvolved retina, anterior chamber, and the optic nerve. In our practice, it is the preferred treatment for melanomas that are non-amenable to standard plaque brachytherapy. Since late 2010, we have treated more than 40 patients with our robotic linear accelerator. This case-based technical note outlines the technique used at the University of Montreal, Montreal, Canada.

## Introduction

Melanoma (most frequently involving the choroid) is the most common primary intraocular cancer [1]. Intraocular tumors remain rare. In Canada, the incidence of primary intraocular tumors has been stable over the last 20 years at 0.7-1 per 100,000 persons per year [2]. This represents 385 intraocular tumors per year in Canada — 80 in Quebec — the majority of which will be seen at our institution.

Although at one time controversy existed as to the role of enucleation for medium-sized choroidal melanomas, the results of Collaborative Ocular Melanoma Study (COMS) clarified that organ preservation can be attempted without impact on overall survival [3]. Despite the fact that patients in the COMS trial were all treated with Iodine 125 plaque brachytherapy, most groups have taken these results to support other forms of focal radiation. In Canada, in addition to I125 brachytherapy, treatment is currently available with Ru106 brachytherapy [4], Au198 brachytherapy [5], proton therapy [6], and various forms of stereotactic radiosurgery/radiotherapy [7].

This case-based technical review outlines the University of Montreal's approach to treating choroidal tumors near or encroaching on the optic nerve. This report follows the guidelines of our local ERB and consent was obtained from the patient whose case is used to illustrate our technique. Herein we detail our immobilization, treatment planning, and treatment delivery protocols.

## Technical report

### Case illustration 

A 69-year-old presented with worsening of intermittent visual symptoms and heaviness in his left eye. On examination, he had a 12 x 12 x 8.6 mm mushroom-shaped pigmented tumor overlying the optic nerve (AJCC stage T2a). The eyesight in the left eye was 20/20 -2. Chest x-ray and abdominal CT scan did not reveal any evidence of distant metastases. As per our usual practice, the patient was referred for stereotactic radiotherapy using the Cyberknife (Accuray, Sunnyvale, CA) — 60 Gy in 10 daily fractions of 6 Gy. 

### Methods 

Immobilization, Simulation, and Planning

The patient undergoes a 1.5T planning MRI with a 20 channel head coil (Magnetom Aera, Siemens Healthcare, Erlangen, Germany). Focused sequences are obtained with the patient fixing a dot within the coil (placed in the approximate position of the light used in our immobilization device); this facilitates image co-registration but is not mandatory. Three sequences are obtained, a thin slice T2 2D Turbo Spin Echo (TSE, “DIXON” on our Siemens unit), a 3D 1 mm isotropic T2 series (“SPACE” on our Siemens unit), and a gadolinium-enhanced 3D T1 isotropic series (“VIBE”). The patient is then immobilized supine in a thick (3.2 mm) thermoplastic mask with Kevlar reinforcement, a cutout for the eyes (in patients unable to see with the involved eye, immobilization is based on the seeing eye), and a wide base to support the camera system. The camera system is part of a custom immobilization device which provides a light for the patient to fix, the position of which can be recorded and reproduced. The camera system allows for monitoring of patient compliance. The position of the iris is marked on a transparency overlaid on the screen linked to the monitoring camera. Simulation CT is acquired with 2 mm thick slices every 1 mm. The field of view is sufficient to visualize the entire immobilization device.

In the planning system (Multiplan version 4.5.0, Accuray, Sunnyvale, CA), CT and both MRI sequences are manually co-registered using the insertion and the optic nerve and lens as principal landmarks.

The gross tumor volume (GTV) is segmented using both MRI sequences and fundus schema. The dimensions of the contoured volume are checked in relation to those measured on ocular ultrasound. A 2 to 2.5 mm planning target margin (PTV) is added, which is trimmed where it obviously extends beyond the sclera. Organs at risk contoured include: ipsilateral lens, ipsilateral optic nerve (this is contoured with a small 1-2 mm gap at the nerve insertion in the globe), ipsilateral lachrymal gland, contralateral eye, immobilization device, and oral cavity. A shell structure is created 1.5 mm beyond the GTV. Collimator selection is a compromise between dose conformity and the treatment duration. The metal parts of the immobilization are blocked. In each case, the contralateral eye and oral cavity are either blocked or spared via strict optimization criteria. The plan is optimized so that the PTV is covered by at least 95% of the prescription dose (typically with the 65-75% isodose volume). The conformity index (nCI) is kept below 1.5. The lachrymal gland is optimized to a mean dose of less than 30 Gy. The entire contralateral eye (with a 1 cm margin) is kept below 2 Gy. When possible, the ipsilateral lens is kept under 2 Gy. A very steep gradient is created at the optic nerve in order to reduce the dose as much as possible without underdosing the PTV. Typical outcomes of the optimization process are noted in Table 1.

**Table 1:** Dosimetric parameters from 20 consecutive patients.

Parameter	Value
MU (#, +/- SD)	23329 +/- 5463
Number of beams (#, +/- SD)	76 +/- 30
Collimator (median, range)	10mm (7.5-12.5mm)
Estimated time per fraction (median, +/- SD)	36min +/- 9min
GTV	
Mean volume (median, +/- SD)	0.23cm3 +/- 0.15cm3
Min dose (median, +/- SD)	7010cGy +/- 280cGy
Max dose (median, +/- SD)	8571cGy +/- 288cGy
Mean dose (median, +/- SD)	7861cGy +/- 260cGy
PTV	
Mean vol (median, +/- SD)	1.37cm3 +/- 0.47cm3
Min dose (median, +/- SD)	5734cGy +/- 191cGy
Max dose (median, +/- SD)	8571cGy +/- 288cGy
Mean dose (median, +/- SD)	7119cGy +/- 219cGy
Ipsilateral lens	
Max dose (median, +/- SD)	295cGy +/- 461cGy
Mean dose (median, +/- SD)	181cGy +/- 214cGy
Ipsilateral lachrymal gland	
Max dose (median, +/- SD)	2073cGy +/- 2172cGy
Mean dose (median, +/- SD)	1267cGy +/- 1144cGy
Optic nerve (as segmented)	
Max dose (median, +/- SD)	5812cGy +/- 1171cGy
V30 (median, +/- SD)	0.1cm3 +/- 0.06cm3

**Figure 1:**
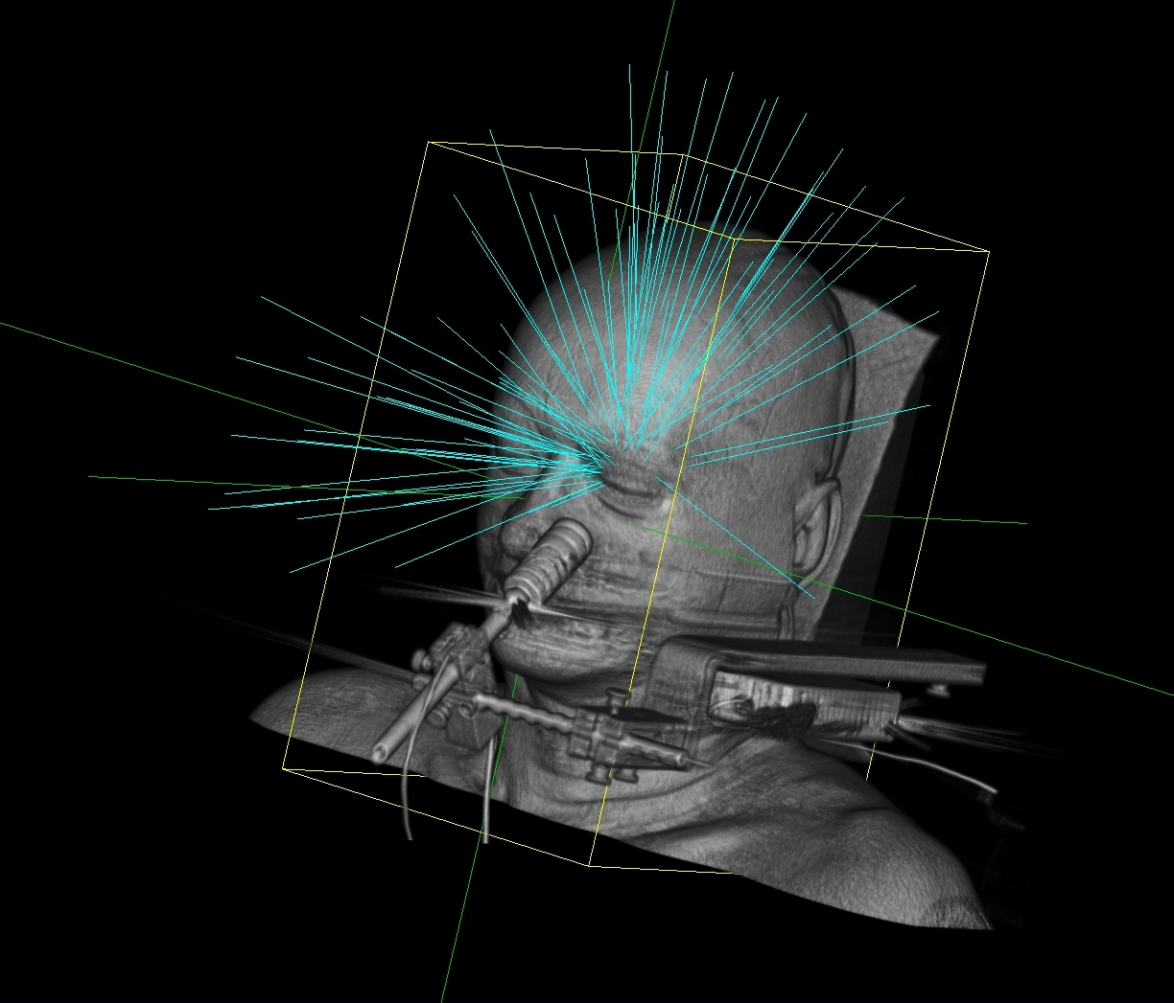
Non-coplanar beam arrangement

The patient in this report was treated with 86 non-coplanar beams with a single 10 mm collimator (Figure 1). The maximum dose to the nerve, lachrymal gland, and ipsilateral lens were 57 Gy, 11 Gy, and 3.9 Gy. The maximum dose to the GTV was 85.7 Gy, and 2719 MU were required to deliver each 6 Gy fraction. With our 1000 MU/min Cyberknife VSI, each fraction thus required approximately 20 minutes to deliver.

Quality Assurance and Delivery 

The original dose was calculated considering tissue heterogeneity using a ray tracing algorithm. An independent monitor unit calculation was used to verify the plan (RadCalc 6.2, Lifeline Software Inc, Austin TX).

A dry run is delivered with the mask, and the immobilization device is performed to identify any potential collisions prior to the first fraction.

The fractions are delivered daily. During treatment, the position of the iris is monitored to be within the markings taken at simulation. Typically the treatment is delivered in one-minute increments between which the patient can rest his eyes. A mid-treatment resimulation is used to confirm the reproducibility of the eye position (both simulation scans are co-registered based on bony anatomy of the cranium, and the position of the eye is compared).

### Results 

**Figure 2:**
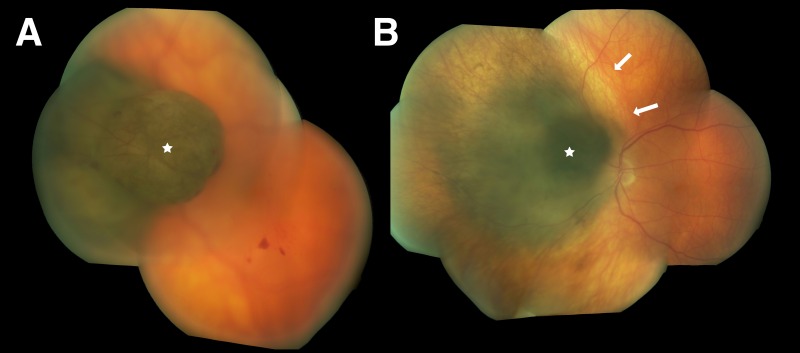
Fundoscopy A: Pigmented mushroom-shaped (star) choroidal melanoma obscuring the optic nerve, measuring 12 x 12 x 8.6 mm in thickness. B: One year after Cyberknife treatment, the tumor has markedly regressed (star). Note the rim of chorioretinal atrophy surronding the tumor (arrows).

The treatments of our illustrative patient were uneventful. As per our clinical practice, the patient received adjuvant intraocular injections of bevacizumab as a prophylaxis against radiation retinopathy [8]. In the two years following radiation, he received eight quarterly injections. Twenty-two months following diagnosis, the tumor was flat and without evidence of local or distant activity (Figure 2). The patient was free of significant radiation retinopathy, and, despite the high dose to a short segment of the optic nerve, maintained a visual acuity of 20/50 -2 in the left eye.

## Discussion

In recent decades, radiosurgery has been used for the treatment of selected cases of ocular melanoma. Treatments have been typically delivered using a single fraction on a Gamma Knife unit with retrobulbar anesthesia [9]. Reports using the CyberKnife platform are less common with the largest experience being from Munich where patients are treated with a regimen analogous to that used on the Gamma Knife (single fraction using retrobulbar anesthesia) [10]. In the German series of more than 200 patients, the estimated five-year local failure rate was approximately 30%, and 30% of patients retained serviceable eyesight (defined as 6/20 or better). These results may be a reflection of patient selection (which included large tumors) rather than the treatment regimen.

The non-invasive nature of the CyberKnife allows consideration of fractionated regimens delivered without any anesthesia [11]. Our technique affords an efficient means of treating ocular melanoma on this robotic radiosurgery platform. The non-coplanar beams afford a very steep dose drop off. Dosimetric comparisons, even when performed on the same patients, are fraught with bias. This being said, the low doses to the anterior chamber of our patients are amongst the lowest for any published radiation technique [7, 12]. Our technique can be further refined. The mid-treatment re-simulation may not be necessary. The PTV margins used may be excessive if they aim only to account for immobilization of the eye and targeting accuracy (manuscript in preparation) and not uncertainties in image registration or tumor definition. For selected patients with lateral tumors, there may also be a benefit to optimizing gaze in order to better spare the lachrymal gland.

The dose regimen used at our institution builds on experience at McGill University treating juxtapapillary choroidal melanoma with conventional 2 Gy fractions using D-shaped Co-60 beams [13]. In comparison to 70 Gy in 35 fractions, our dose regimen of 60 Gy in 10 fractions (invariable with a higher minimum dose to the GTV) is significantly hypofractionated. On the other hand, a 10-fraction regimen is amongst the more protracted regimen amongst the various published experiences with radiosurgery, stereotactic linac radiation, or proton therapy [14].  Comparisons between retrospective series from different eras with heterogeneous tumors offer limited insight into which regimen affords the best therapeutic ratio. In some cases, the choice is limited by the device (single fraction treatments are most common using invasive immobilization on the Gamma Knife) or logistics (in some cases, limited access to a proton-beam line can limit treatment duration). Our experience is no different in that we have yet to have a local failure in a patient with a small or medium-sized tumor — leading credence that 6 Gy fractions are sufficient to overcome the potentially large shoulder on the survival curve of uveal melanoma cell lines [15].

In 1996, Dr. Fine published an editorial provocatively titled “No One Knows the Preferred Management for Choroidal Melanoma” [16]. At the time, he was referring to the controversy opposing enucleation to eye-preserving treatments. The same statement would be accurate today for juxtapapillary tumors, but may oppose notched plaque brachytherapy, proton therapy, radiosurgery, and varied regimens of photon stereotactic radiation. Unfortunately, no treatment of juxtapapillary tumors will completely spare the patient from the risks of radiation retinopathy, optic neuropathy, neovascular glaucoma, iatrogenic cataract, tumor failure, or metastases (the visual outcome in our illustrative case was better than average in our series of juxtapupillary tumors).

In our review of the options available to our patients, it is our decision to shy away from notched plaques (which would often require additional treatment, typically transpupillary thermotherapy [17], to avoid tumor failure at the optic nerve margin). We currently offer our patients:

• Ru106 plaque brachytherapy when a tumor of ≤ 5mm in height can be treated in less than seven days within the 50% isodose line of a plaque held in our inventory (typically 85 Gy to the tumor apex)

• I125 plaque brachytherapy for tumors 16 mm or less in diameter, up to 10-12 mm in height and ≥2 mm from the optic nerve (typically 85 Gy to the tumor apex, dose which can be lowered for the thickest tumors)

• Fractionated stereotactic radiation for tumors <2 mm from the optic nerve, 60 Gy in 10 daily fractions

## Conclusions

Stereotactic robotic radiotherapy for ocular melanoma represents an attractive alternative in the conservative management of ocular melanoma. In our practice, it is the preferred means of treating juxtapapillary tumors. As with other radiation techniques, its potential benefits or shortcomings will likely elicit debate fueled as much by opinion as by fact.
